# Atypical Q Fever in US Soldiers

**DOI:** 10.3201/eid1308.070218

**Published:** 2007-08

**Authors:** Joshua D. Hartzell, Suzette W. Peng, Robert N. Wood-Morris, Dennis M. Sarmiento, Jacob F. Collen, Paul M. Robben, Kimberly A. Moran

**Affiliations:** * Walter Reed Army Medical Center, Washington DC, USA; † Uniformed Services University of the Health Sciences, Bethesda, Maryland, USA; ‡ 28th Combat Support Hospital, Baghdad, Iraq

**Keywords:** Q fever, *Coxiella burnetii*, cholecystitis, acute respiratory distress syndrome, military, dispatch

## Abstract

Q fever is an emerging infectious disease among US soldiers serving in Iraq. Three patients have had atypical manifestations, including 2 patients with acute cholecystitis and 1 patient with acute respiratory distress syndrome. Providers must be aware of Q fever’s signs and symptoms to avoid delays in treatment.

Q fever, caused by infection with *Coxiella burnettii,* is an emerging infectious disease among US soldiers deployed to Iraq and Afghanistan; >30 cases have been reported (*1*–*3*). We describe 3 cases of Q fever in soldiers treated from July through December 2006 at Walter Reed Army Medical Center (WRAMC).

## The Patients

In December 2006, 1 week after returning from Iraq, a 22-year-old white male Army National Guard member was seen at a New Hampshire hospital, with flulike symptoms, pleuritic chest pain, and mild abdominal pain. His initial examination noted temperature of 38.3°C, leukocytes 3.3 × 10^9^ cells/µL (normal 4.5–10.5 × 10^3^ cells/μL), platelets 121 × 10^3^ cells/μL (normal 150–450 × 10^3^ cells/μL), aspartate aminotransferase (AST) 144 IU/L (normal 15–46 IU/L), and alanine aminotransferase (ALT) 154 IU/L (reference 11–66 IU/L). He was admitted and treated with ceftriaxone and azithromycin. Although his fever decreased within 48 h, he had persistent abdominal pain, worsening liver function test results (AST 779, ALT 993, alkaline phosphatase 269 U/L [reference 38–126 U/L]), and increasing shortness of breath. An ultrasound examination of the right upper quadrant showed hepatosplenomegaly and a thickened gall bladder wall without evidence of cholelithiasis. Despite initially normal chest radiographic results, a repeat radiographic examination showed bilateral pulmonary infiltrates. Ceftriaxone therapy was discontinued, pipercillin/tazobactam therapy was started, and azithromycin was continued. General surgery stated that the patient had a nonsurgical abdomen. After consultation with WRAMC, the patient was given a dose of doxycycline and gentamicin before being transferred to a New Hampshire medical center. Blood cultures and serologic tests for Epstein Barr virus and cytomegalovirus were pending. A computed tomographic (CT) examination of the chest, abdomen, and pelvis (Figure, left panel) showed gall bladder wall thickening (10 mm) without ductal dilatation, hepatosplenomegaly, and bilateral ground glass pulmonary infiltrates. Serologic tests were negative for hepatitis B and C. Thick and thin smears were negative for parasitic disease. Despite the findings on CT scan, the patient began to improve clinically and had resolution of abdominal pain and shortness of breath. He was transferred to WRAMC, where he continued to improve. Pipercillin/tazobactam was discontinued, but doxycycline was continued. A presumptive diagnosis of Q fever was made, and he was discharged to complete a 14-day course of doxycycline. Serologic tests for *C. burnettii* were positive with a phase 2 immunoglobulin M (IgM) titer of 256 (negative <1:64), phase 2 IgG titer of 128 (negative <16), and negative phase 1 serologic results. A month later he felt well and had normal liver function test results. No exposure factors were identified.

**Figure:**
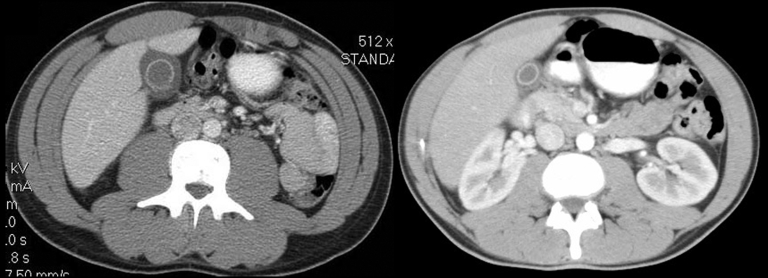
Computed tomographic scans of abdomens of 2 patients with inflammation of the gallbladder.

The second case occurred in December 2006, when a previously healthy 24-year-old male Army National Guard member was admitted to the 28th Combat Support Hospital (CSH) in Baghdad, Iraq, with flulike symptoms, mild nausea, and a dry, 10-day cough. At admission, his temperature was 40.2°C, but his other vital signs were normal. He had mild epigastric tenderness to palpation; otherwise, examination results were normal. Laboratory results included leukocytes 3.9×10^3^ cells/μL, platelets of 130×10^3^ cells/μL, alkaline phosphatase 104 U/L, AST 824 U/L, ALT 786 U/L, total bilirubin 1.2 mg/dL (reference 0.2–1.3 mg/dL), and gamma glutamyl transferase (GGT) 97 (reference 12–58). Initial erythrocyte sedimentation rate was within normal limits at 18 mm/hr (reference <20 mm/h). Results of blood cultures, monospot, and hepatitis B, C, and HIV screens were negative. A CT scan showed diffuse enhancement of the gallbladder with gallbladder wall thickening (Figure, right panel). A small amount of pericholecystic fluid was seen, but no distension of the gallbladder or gallstones were noted. These findings prompted a general surgery evaluation for acute cholecystitis, but their examination results were not consistent with this diagnosis. Given the patient’s flulike symptoms and laboratory abnormalities, the diagnosis of Q fever was considered. The patient had initially been treated with doxycycline and metronidazole, but metronidazole was discontinued when his physical examination results remained benign. His fever curve decreased within 2 days of receiving doxycycline. He was transferred out of theater to Landstuhl Regional Medical Center in Germany for further evaluation. Q fever was confirmed with *C. burnettii* serum titers of 2,048 for phases 1 and 2 IgM. He improved with doxycycline, 100 mg twice a day for 14 days, and was subsequently returned to duty. No exposure factors were identified.

The third case occurred in July 2006 in a 34-year-old female active duty soldier with a history of asthma. She was seen at the troop medical clinic in Baghdad, Iraq, with flulike symptoms. She was given symptomatic treatment and released but returned with altered mental status, shortness of breath, and abdominal pain. A CT scan of her chest showed a left lower lobe infiltrate and bilateral pleural effusions. An ultrasound examination of the right upper quadrant showed no abnormalities. She was transferred to the 10th CSH in Baghdad for further care. She remained febrile (39.8°C) and tachycardic and required 4 L/min of oxygen via nasal cannula to maintain an oxygen saturation of 96%. Results of laboratory tests conducted at the time of admission were unremarkable except for a mild transaminitis (AST 139 and ALT 96). She was treated with levofloxacin, 500 mg per day intravenously, for suspected pneumonia. She had rapid worsening of her respiratory status over the next 8 hours and required intubation. Antimicrobial drug coverage was broadened to include piperacillin/tazobactam 3.375 parenterally every 6 hours; solumedrol was added, given her history of asthma. She was evacuated to Landstuhl Regional Medical Center in Germany. A bronchoscopy was performed, but results were unremarkable. Her chest radiographs showed progression to acute respiratory distress syndrome (ARDS), and arterial blood gas testing showed partial pressure of arterial oxygen to be 50–60 mm Hg. Blood, sputum, and urine cultures were negative. Doxycycline was prescribed for possible Q fever. She improved and was evacuated to WRAMC, where she was afebrile (37.2°C) at admission. Her pulmonary status improved quickly, and she was extubated. She was discharged and completed 14-day courses of levofloxacin and doxycycline. Her serologic test results were positive for Q fever with phase 2 IgM titer of 1,024. No exposure risks were identified.

## Conclusions

Fever, pneumonia, and/or hepatitis are the most common signs of acute infection with Q fever (*4*,*5*). In those in whom chronic disease develops, infective endocarditis is the initial condition in >70% of cases. Asymptomatic infection may occur in >50% of infected patients (*4*,*5*). Despite its typical signs and symptoms, Q fever is known to have a multitude of clinical manifestations. Raoult described >7 distinct presentations (*6*): fever, pneumonia, hepatitis, meningitis, meningoencephalitis, pericarditis, and myocarditis. Parker et al. described >30 clinical syndromes (*4*). This broad variation can result in delayed diagnosis.

Only 12 cases of acute cholecystitis associated with Q fever have been reported in the English medical literature (*7*–*10*). The largest and most detailed description is from a case series by Rolain (*7*), who described 9 patients whose initial sign of Q fever was acute cholecystitis. Clinical data are available for only 1 other case (*8*). The most appropriate treatment for these patients remains a question. For these 10 patients, 6 had cholecystectomy. The remaining 4 and our 2 patients did well with medical management alone. Four of the 6 patients received doxycycline, 1 received ofloxacin, and 1 received no treatment. Q fever is often self-limiting; yet treatment is recommended to shorten duration of symptoms and prevent chronic disease (*5*).

Reina-Serrano recently suggested that patients with Q fever–associated cholecystitis could be managed medically (*8*). Two of our patients had evidence of cholecystitis on imaging studies but did not have evidence of peritonitis on physical examination. Our 2 patients with radiographic cholecystitis responded quickly to doxycycline. We propose that for patients with acute acalculous cholecystitis and a high suspicion for Q fever, doxycycline be given empirically. The patients’ clinical response should be evident within 48 hours and surgery may be avoided. If a patient has gallstones or acute abdominal pain, a standard approach for treating acute cholecystitis should be followed.

The third patient in our series progressed to ARDS, which has been reported, albeit rarely, with Q fever (*1*,*4*,*11*,*12*). More typically, pneumonia secondary to acute Q fever infection results in a dry to productive cough, pleuritic chest pain, and focal or bilateral infiltrates on chest radiographs (*6*).

Our patients denied having typical risk factors, including exposure to livestock or consumption of local meat or dairy products. However, direct exposure to such products is not necessary (*1*,*4*,*5*). We agree with Anderson et al., who suggested that providers strongly consider adding doxycycline to the treatment regimen for deployed soldiers with severe pneumonia (*1*).

Q fever is a Category B biologic agent and must be considered as a potential threat to deployed soldiers (*13*). The most likely mode of attack would be aerosolization; given the low dose required for infection (1–10 organisms), multiple cases would follow. We considered bioterrorism unlikely, given the limited number of clinically symptomatic cases and the lack of a cluster of cases.

Q fever continues to be a threat to deployed US soldiers in Southwest Asia. Lack of knowledge about it can delay diagnosis and treatment. It should be considered in the differential diagnosis of any deployed or recently deployed soldier with a febrile illness, especially when hepatitis or pneumonia is present.
